# Receptor for Activated Protein Kinase C: Requirement for Efficient MicroRNA Function and Reduced Expression in Hepatocellular Carcinoma

**DOI:** 10.1371/journal.pone.0024359

**Published:** 2011-09-15

**Authors:** Motoyuki Otsuka, Akemi Takata, Takeshi Yoshikawa, Kentaro Kojima, Takahiro Kishikawa, Chikako Shibata, Mutsuhiro Takekawa, Haruhiko Yoshida, Masao Omata, Kazuhiko Koike

**Affiliations:** 1 Department of Gastroenterology, Graduate School of Medicine, The University of Tokyo, Tokyo, Japan; 2 Department of Cell Signaling and Molecular Medicine, Research Institute of Environmental Medicine, Nagoya University, Nagoya, Japan; University of Hong Kong, Hong Kong

## Abstract

MicroRNAs (miRNAs) are important regulators of gene expression that control physiological and pathological processes. A global reduction in miRNA abundance and function is a general trait of human cancers, playing a causal role in the transformed phenotype. Here, we sought to newly identify genes involved in the regulation of miRNA function by performing a genetic screen using reporter constructs that measure miRNA function and retrovirus-based random gene disruption. Of the six genes identified, RACK1, which encodes “receptor for activated protein kinase C” (RACK1), was confirmed to be necessary for full miRNA function. RACK1 binds to KH-type splicing regulatory protein (KSRP), a member of the Dicer complex, and is required for the recruitment of mature miRNAs to the RNA-induced silencing complex (RISC). In addition, RACK1 expression was frequently found to be reduced in hepatocellular carcinoma. These findings suggest the involvement of RACK1 in miRNA function and indicate that reduced miRNA function, due to decreased expression of RACK1, may have pathologically relevant roles in liver cancers.

## Introduction

MicroRNAs (miRNAs) are short (20-23-nt), endogenous, single-stranded RNA molecules, that regulate gene expression and control physiological and pathological processes, such as development and cancer. Mature miRNAs and Argonaute (Ago) proteins form the RNA-induced silencing complex (RISC), a ribonucleoprotein complex that mediates post-transcriptional gene silencing [1] and then, complementary base-pairing of miRNAs guides the RISC to target messenger mRNAs, which are subsequently destabilized and sequestered from the translational machinery by Ago proteins [2]–[5]. Although insights into the regulatory function of miRNAs are beginning to emerge, their mechanisms of action and the genes involved in miRNA pathway have not yet been fully determined [6], [7].

A large body of evidences suggests that the multigene regulatory capacity of miRNAs is dysregulated and exploited in cancer. Although several miRNAs are upregulated in specific tumors [8], a global reduction of miRNA abundance appears a general trait of human cancers, playing a causal role in the transformed phenotype [9], [10]. In fact, the enzymes and cofactors involved in miRNA processing pathways may be targets of genetic disruption, further enhancing cellular transformation [9]. Moreover, the disruption of Dicer in mice promotes hepatocarcinogenesis [11] and the truncating mutations in TARBP2, which causes a defect in the processing of miRNAs, were identified in sporadic and hereditary colon carcinomas [12].

Retroviral insertion-mediated random gene disruption can be used to generate null alleles, resulting in diminished endogenous gene expression [13]. The use of such retroviral integration methods, combined with appropriate reporter constructs, has provided an efficient, comprehensive gene screening method [14], [15]. In this study, we sought to identify new genes involved in miRNA function and determine its role in live cancers. To this end, we established reporter cell lines in which cellular miRNA function could be assessed by expression of a drug resistance gene. Using these cell lines and a random gene disruption method, we identified genes that have not previously been implicated in the regulation of miRNA function. We subsequently determined the role of one of these genes, RACK1, which encodes “receptor for activated protein kinase C” (RACK1), in miRNA function, as well as its expression in liver cancers. Collectively, our data suggest the potential involvement of RACK1 in pathological processes.

## Results

### Identification of the genes required in the miRNA pathway by random gene disruption

**Figure 1:**
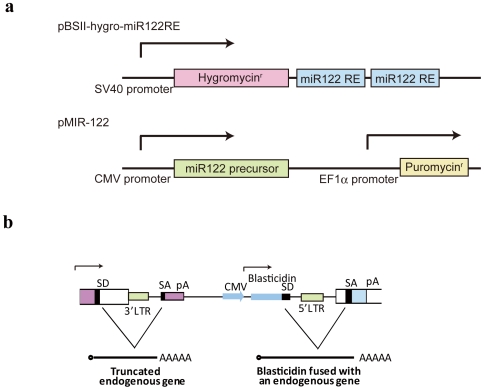
Constructs used in the study. **A,** Reporter and miR122 precursor-expressing constructs. A construct containing an SV40 promoter-driven hygromycin resistance gene with two tandem miR122-responsive elements (miR122 RE) in its 3′-UTR was used to assess miRNA function. To express miR122, a construct carrying a CMV promoter-driven miR122 precursor was used in conjunction with puromycin selection. **B,** pDisrupt vector structure and gene products resulting from viral integration. Splicing occurs between the SD (splicing donor) and SA (splicing acceptor) sites (i.e., between the 3′-end of the endogenous gene exon and the retroviral SA site, as well as between the retroviral SD at the 3′-end of the blasticidin gene and the endogenous SA site at the 5′-end of the downstream gene exon). pA, polyadenylation signal.

To identify genes involved in miRNA pathways especially in liver cells, we constructed a reporter carrying a hygromycin resistance gene with two miR122-responsive elements in its 3′-UTR (Fig. 1A). We chose miR122 because it is the most abundant and tissue-specific miRNA in the liver [16]. Binding of miR122 reduces the expression of this hygromycin resistance gene in this construct. However, if miR122 function is impaired by the disruption of genes that are important for miRNA signaling, hygromycin resistance gene expression increases. Cells carrying such disrupted genes will therefore survive hygromycin treatment. Additionally, to enhance the effects of miR122, we co-transfected a miR122 precursor-expressing plasmid (Fig. 1A) with the reporter construct and selected monoclonal cells containing both constructs to minimize the effects of their random integration. After infection with retroviruses carrying a blasticidin resistance gene to produce random gene disruption in the selected reporter cells, cells surviving hygromycin treatment were harvested. The disrupted genes in the surviving cells were identified by 3′ RACE. We infected ∼10^6^ Huh7-pBS-Hygro-miR122 cells established from Huh7 cells and obtained ∼10^4^ clones with random gene disruptions (confirmed by resistance to blasticidin) (Fig. 1B). After hygromycin selection, ten clones in which miRNA function was apparently impaired were obtained. In these ten clones, six disrupted genes were successfully identified (Table 1). One of them, RACK1 (also known as GNB2L1), appeared in duplicate and was found to be disrupted in two clones.

**Table 1:** List of genes identified in this screening.

Gene ID	Gene symbol	Gene title	Known function	Also known as
100288263	LOC100288263	hypothetical protein	Unknown	
10399	GNB2L1	guanine nucleotide binding protein (G	Translation	RACK1, PIG21
		protein), beta polypeptide 2-like 1		
10055	SAE1	SUMO1 activating enzyme subunit 1	Sumoylation	AOS1, SUA1, UBLE1A
10399	GNB2L1	guanine nucleotide binding protein (G	Translation	RACK1, PIG21
		protein), beta polypeptide 2-like 1		
6228	RPS23	ribosomal protein S23	Ribosome 40S	FLJ35016
6135	RPL11	ribosomal protein L11	Ribosome 60S	DBA7, GIG34
5358	PLS3	plastin 3	Actin binding	T-plastin

### Requirement of RACK1 for microRNA function

Of the genes identified, RACK1 was found to be disrupted in two independent clones (Table 1). RACK1 was previously identified as the binding partner of eIF6, a ribosome inhibitory protein known to prevent proper assembly of the 80S ribosome, and contributes to miRNA silencing by associating with RISC [17]–[19]. However, the requirement for eIF6 in miRNA function is controversial because other models, in which miRNA silencing is mediated by Ago2 (eIF2C2) and an interaction with eIF4E [20], or by GW182 [21], have more recently been described.

**Figure 2:**
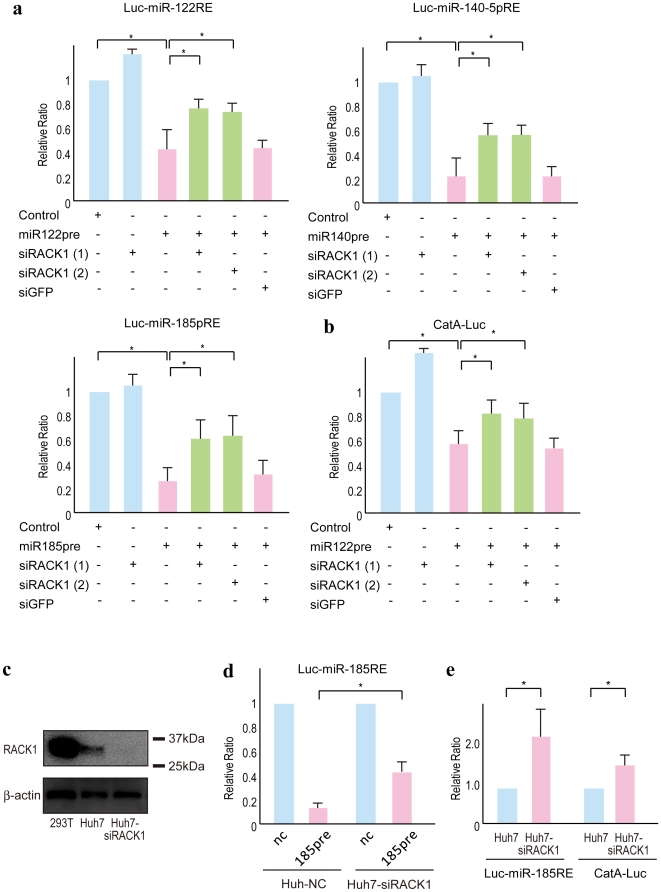
RACK1 is required for miRNA function. **A,** Overexpression of miR122, miR140, and miR185 precursors suppressed activity of the corresponding reporters. RACK1 knockdown partially blocked these effects. miRNA reporter plasmids were transfected, with or without the corresponding miRNA precursor- and two types of siRACK1-expressing plasmids (siRACK1 (1) and (2)), into Huh7 cells. Values were normalized to those obtained from cells transfected with a miRNA precursor-non-expressing control vector, which were set to 1. Data represent the mean ± SD of three independent experiments. siGFP was used as a control, and it had no effect. Similar results were obtained using PLC/PRF/5 cells. **B,** CatA-Luc plasmids, which contain endogenous miR122 target sites derived from the CAT1 gene in its 3′-UTR, were transfected, with or without miR122 precursor- and siRACK1-expressing plasmids, into Huh7 cells. Data were generated and are presented as described in (A). Similar results were obtained using PLC/PRF/5 cells. **C,** Confirmation of the efficient knockdown of RACK1 expression in stable RACK1-knockdown Huh7 cells. 293T cell lysates were used as a positive control. **D,** miRNA function is impaired in stable RACK1-knockdown cells. miRNA185 reporter plasmids were transfected, with or without miR185 precursor-expressing plasmids, into control and RACK1-knockdown cells. Data were generated and are presented as described in (A). **E,** Endogenous miRNA function is impaired in RACK1-knockdown cells. miRNA185 reporter plasmids and CatA-Luc plasmids were transfected without miRNA precursor-expressing plasmids into control and RACK1-knockdown cells to assess endogenous miRNA function. Values were normalized to those obtained from control cells, which were set to 1. Data represent the mean ± SD of three independent experiments. *, p<0.05.

To confirm the requirement for RACK1 in miRNA function, we measured miRNA activity using a reporter assay involving transient expression of an siRACK1 construct and an miRNA precursor-overexpression plasmid. Transient knockdown of RACK1 reduced the function of three miRNAs: miR122, miR140, and miR185 (Fig. 2A). To examine these effects using a natural 3′-UTR containing miR122 binding sites, we used a CatA-Luc reporter that carried the 3′-UTR of the CAT1 (cationic amino acid transporter 1) gene and a luciferase gene [22]. The CAT1 3′-UTR contains three predicted miR122 binding sites [22]. The requirement for RACK1 in miRNA function was confirmed using this reporter construct (Fig. 2B).

Next, we established stable RACK1-knockdown Huh7 cells (Fig. 2C) and compared the effects of miRNAs in control and RACK1-knockdown cells. Consistent with the results of the transient transfection assays, RACK1-knockdown cells showed weaker miRNA mediated-inhibition of target gene expression in a reporter system involving miR185 precursor-expressing plasmids and its reporter constructs (Fig. 2D). To measure changes in endogenous miRNA function in RACK1-knockdown cells, control and RACK1-knockdown cells were transfected with reporter constructs specific for several miRNAs and luciferase activity was then measured. We confirmed the inhibition of endogenous miRNA function in RACK1-knockdown cells (Fig. 2E). These results suggest that RACK1 is indeed required for miRNA function.

### RACK1 may function after miRNA maturation but before the expression-inhibitory machinery

**Figure 3:**
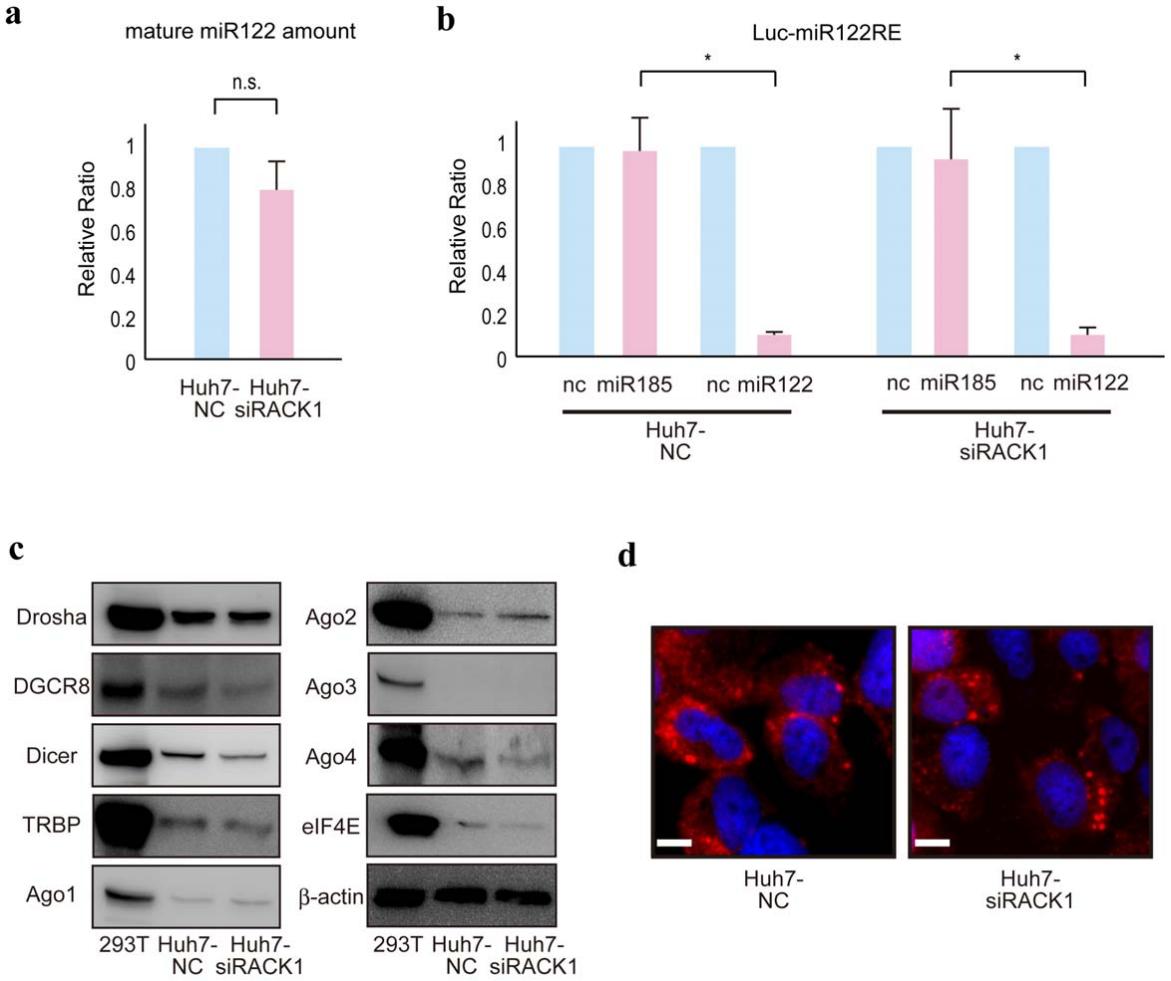
RACK1 functions after miRNA maturation and before the silencing machinery. **A,** miRNA maturation was not impaired in RACK1-knockdown cells. Total RNA was isolated from control and RACK1-knockdown cells. Levels of mature miR122 were measured and normalized to the level of U6 snRNA. Relative ratios were calculated by adjusting the value for each miRNA in control cells to 1. Data represent the mean ± SD of six independent experiments. **B,** Artificial synthetic miRNA oligonucleotides function appropriately in RACK1-knockdown cells. Control Huh7 cells and RACK1-knockdown cells were transfected with miR122 reporter plasmids with or without synthetic corresponding miR122 oligonucleotides and non-corresponding miR185 oligonucleotides (to verify specificity). Values were normalized to those obtained from the cells transfected with control synthetic oligonucleotides, which were set to 1. Data represent the mean ± SD of three independent experiments. *, p<0.05. Similar results were obtained using PLC/PRF/5 cells. **C,** Expression of proteins involved in the miRNA pathway was normal in RACK1-knockdown cells. 293T cell lysates were used as a positive control. **D,** Localization and expression of P-bodies were normal in RACK1-knockdown cells. Control and RACK1-knockdown cells were immunostained for the p-body marker GW182. Scale bar, 50 µm.

To identify the point at which RACK1 enhances miRNA function, mature miRNA levels were first measured in stable RACK1-knockdown cells. Levels of endogenous mature miR122, miR22, miR140-5p, -3p, and miR185, which are expressed at relatively high levels in liver cells [16], were comparable in control and RACK1-knockdown cells (Fig. 3A and Figure S1). This suggests that RACK1 may not be involved in miRNA maturation. We next showed that overexpression of artificial synthetic miRNA oligonucleotides replicated normal miRNA-mediated inhibition of gene expression in RACK1-knockdown cells (Fig. 3B). These results suggest that, in vivo, RACK1 may function after miRNA maturation, but before the expression-inhibiting machinery in the natural miRNA pathway, although the finding that the synthetic mature miRNAs were functional even in Ago2-knockdown cells (Figure S2A, B) indicates that their function might be Ago2-independent. Additionally, the expression of Drosha, DGCR8, Dicer, TRBP, Ago1, Ago2, Ago3, Ago4, and eIF4E, all of which are known to be involved in the miRNA pathway, were almost similar in control and RACK1-knockdown cells (Fig. 3C). Furthermore, the localization and number of the p-bodies, which may be involved in miRNA-mediated silencing [23], [24], were not markedly affected by RACK1-knockdown (as determined by staining for the intracellular marker GW182) [23] (Fig. 3D). These results suggest that RACK1 functions after miRNA maturation, but before mature miRNAs exert their expression-inhibitory effects.

### RACK1 interacts with KSRP and is required for the full recruitment of mature miRNAs to the RISC

**Figure 4:**
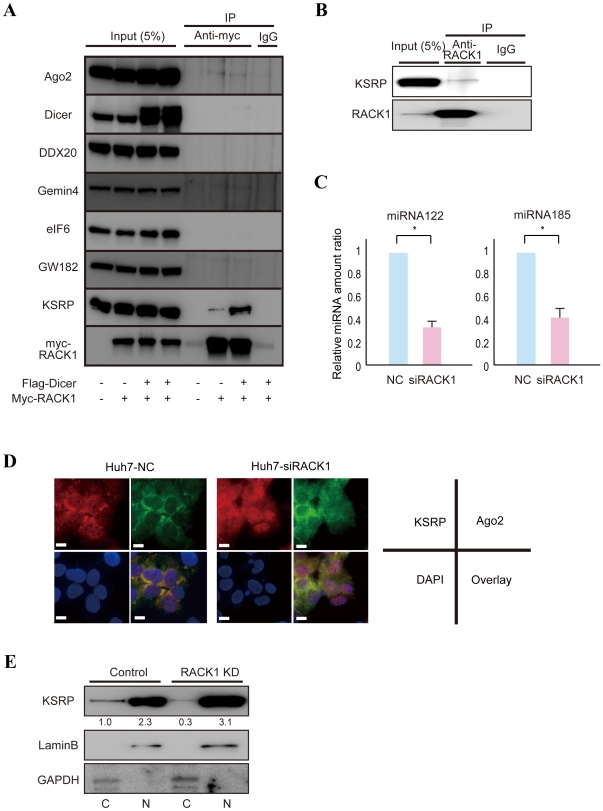
RACK1 binds to KSRP and may be involved in the recruitment of mature miRNAs to the RISC. **A,** Huh7 cells were transiently transfected with a control vector or a myc-tagged RACK1-expressing plasmid with or without a flag-tagged Dicer-expressing plasmid. Myc-tagged RACK1 was immunoprecipitated using anti-myc agarose. Normal mouse IgG was used as a control for immunoprecipitation. Co-precipitated proteins were blotted using antibodies against the indicated proteins. Five percent of the total cell lysates was loaded as “input.” Representative results from two independent experiments are shown. Similar results were obtained using 293T cells. **B,** Endogenous RACK1 in Huh7 cells was immunoprecipitated using anti-RACK1 antibody and Protein A/G Sepharose. Normal mouse IgG was used as a control for immunoprecipitation. Coprecipitated proteins were blotted using antibodies against the indicated proteins. Five percent of the total cell lysate was loaded as “input.” Representative results from two independent experiments are shown. **C,** Levels of mature miR122 and miR185 in Ago2-containing complexes were reduced in RACK1-knockdown cells. Mature miRNA levels were measured in RNA samples isolated from Ago2-containing complexes from control Huh7 (NC) cells and RACK1-knockdown (siRACK1) cells. miRNA levels were calculated as relative ratios. Data represent the mean ± SD of six independent experiments. *, p<0.05. **D,** More KSRP localizes in the nucleus in RACK1-knockdown cells. Intracellular localization of KSRP (red) and Ago2 (green) were examined in control and RACK1-knockdown cells. KSRP was distributed both in the nucleus and cytoplasm, but more KSRP localized in RACK1-knockdown cells. Scale bar, 50 µm. **E,** Greater localization of KSRP to the nucleus in RACK1-knockdown (RACK1 KD) cells was confirmed by Western blotting. Cytoplasmic (C) and nuclear (N) fractions were blotted with anti-KSRP antibody. The numbers below the panel indicate relative KSRP protein levels. GAPDH (a cytoplasm marker) and Lamin B (a nucleus marker) were blotted to confirm the appropriate fractionation.

To determine whether RACK1 interacts with miRNA pathway related-molecules, transiently-transfected myc-tagged RACK1 was first immunoprecipitated. While myc-tagged RACK1 was efficiently precipitated (Fig. 4A), RISC constituent proteins such as Dicer, DDX20 (Gemin3) and Gemin4 [25] were found not to interact with RACK1 (Fig. 4A). GW182 did not interact with RACK1 (Fig. 4A). eIF6, which was previously reported to interact with RACK1 [18] and which may be involved in miRNA silencing [19], did not show an interaction with RACK1 in the present study (Fig. 4A). However, Ago2 weakly interacted with RACK1 (Fig. 4A), as reported recently [26]. Moreover, KH-type splicing regulatory protein (KSRP; also known as KHSRP), a key mediator of mRNA degradation [27], [28] and a component of the Dicer complex that promotes the maturation of a subset of miRNAs [29], interacted with RACK1, especially when Dicer was overexpressed (Fig. 4A and Figure S3). This interaction was also confirmed by immunoprecipitation of endogenous RACK1 (Fig. 4B). The interactions of RACK1 with Ago2 and KSRP were insensitive to RNase A treatment (Figure S4), suggesting that they were not mediated by RNAs. Next, because it seemed that RACK1 functioned after miRNA maturation and before mature miRNA recruitment into the RISC, we measured levels of miRNAs in complexes immunoprecipitated using an anti-Ago2 antibody. Levels of mature miRNAs examined in the Ago2-containing complexes were lower in RACK1-knockdown cells than in control cells (Fig. 4C and Figure S5). To determine the possible causes of impaired mature miRNA loading into Ago2-related complexes in RACK1-knockdown cells, we determined the intracellular localization of KSRP and Ago2 in RACK-1 knockdown cells (Fig. 4D). While KSRP is distributed both in the nucleus and cytoplasm and Ago2 localizes mainly in cytoplasm in control cells, KSRP localizes more in the nucleus in RACK1-knockdown cells (Fig. 4D). The changes in the subcellular localization of KSRP in RACK1-knockdown cells were also confirmed by Western blotting using cell lysates prepared after subcellular fractionation (Fig. 4E). The function of synthetic mature miRNAs was not affected by KSRP knockdown (Figure S6A, B), suggesting that KSRP itself is not required for RISC activity. Thus, these localization changes with RACK-1 may be related to the impaired mature miRNA loading into Ago2 complexes from the KSRP-associated complexes, although, because the binding of KSRP to Ago2 could not be detected in our coimmunoprecipitation study (Figure S7), the precise mechanisms remain to be elucidated. Nonetheless, these results suggest that RACK1 interacts with KSRP and that the recruitment of mature miRNAs into the RISC is impaired in RACK1-knockdown cells.

### RACK1 expression is frequently decreased in liver cancers

**Figure 5:**
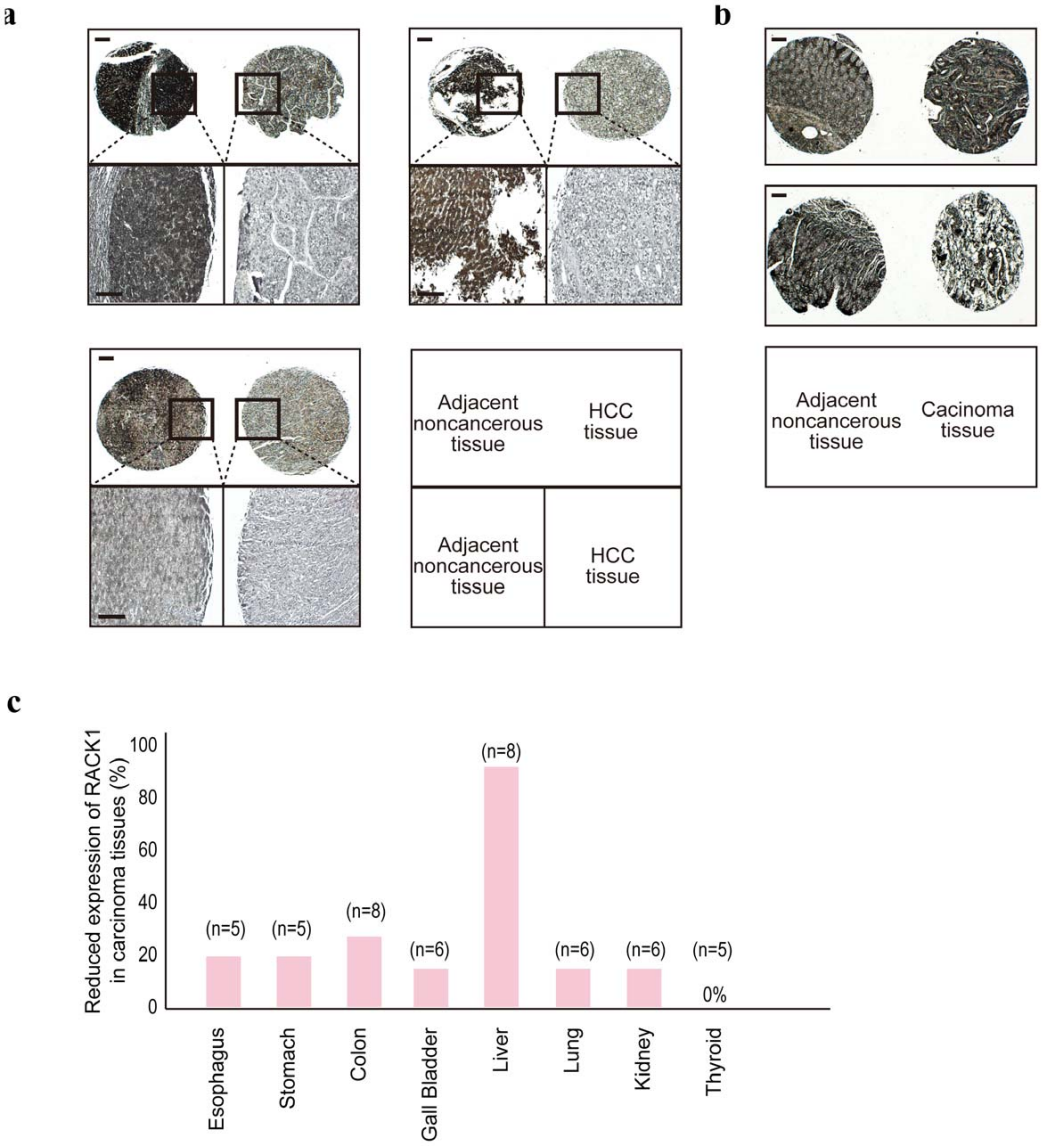
RACK1 expression is reduced in HCC. **A,** Immunohistochemical analysis of RACK1 protein expression in HCC and non-cancerous surrounding tissues. While strong staining was observed in the cytoplasm of hepatocytes in non-cancerous liver tissues (upper left (one panel)), HCC cells were stained more weakly (upper right). Lower panels: magnified images of the highlighted regions in the corresponding upper panels. Three representative cases are shown. Scale bar, 500 µm. **B,** Comparable expression of RACK1 in colon carcinoma tissues (right images) and non-cancerous surrounding tissues (left). Two representative cases are shown. Scale bar, 500 µm. **C,** Comparison of RACK1 staining in cancers and healthy surrounding tissues. Eight types of cancers were examined. Percentages of cases in which RACK1 expression was lower in cancerous tissues than in healthy tissues were calculated. The number of cases of each type of cancer studied is indicated.

Recent results linking reduced global expression of miRNAs and reduced miRNA function with tumorigenesis [12], [30] encouraged us to examine RACK1 expression in cancers. To this end, we determined RACK1 expression in various cancers and in healthy tissues by immunohistochemistry. While RACK1 expression was essentially normal in most cancers, it was frequently reduced in hepatocellular carcinoma (HCC) (Fig. 5A, B, C), consistent with a previous report [31]. KSRP expression levels were relatively higher in liver tissues than in other organs, but no remarkable expression differences between HCC and adjacent tissues were observed (Figure S8A, B). These results suggest that decreased RACK1 expression and, consequently, decreased miRNA function may play an important role in HCC.

## Discussion

Here, we have shown that RACK1 is required for miRNA function through comprehensive gene screening using a random gene disruption method. Our results show that RACK1 interacts with KSRP and plays an important role in recruiting mature miRNAs to the RISC. Additionally, the expression of RACK1 is frequently decreased in HCC and may play a role in its pathogenesis.

RACK1 was initially identified as a major component of active ribosomes [18]. It binds directly to eIF6, which keeps the 40S and 60S ribosomal subunits apart, preventing the formation of a translationally competent complex. RACK1 bridges PKC and eIF6. Subsequent phosphorylation of eIF6 by PKC causes eIF6 to be released, an event that triggers 60S subunit activation. More recently, eIF6 was reported to be a component of a large TRBP-containing complex involved in miRNA-mediated post-transcriptional silencing [19]. However, the importance of eIF6 in miRNA-mediated silencing remains controversial [21]. In this study, we report that, although RACK1 is involved in miRNA-mediated silencing, it did not influence of translational machinery complexes containing eIF6, but instead appears to recruit mature miRNAs to the RISC.

The importance of RACK1 in regulating miRNA function was independently reported during the preparation of this manuscript [26]. Similar to our cases, they reported that RACK1 is required for miRNA function; however, the mechanism they reported differed from ours. They found that RACK1 contributes to the recruitment of RISC to the site of translation through binding with Ago2. While we could not detect the effects of RACK1 on miRNA-mediated translational repression as described above, our results were based on the use of synthesized mature miRNAs, which appear functionally Ago2-independent. Due to the fact that we also detected binding between RACK1 and Ago2, their proposed mechanism remains a possibility [26]. In addition, the importance of these diverse mechanisms might be dependent on different types of miRNAs. For example, the maturation of miRNAs used in our study, i.e., miR122, miR140, and miR185, is KSRP-independent [29]. Taking these points into consideration, in the future it may be necessary to determine if RACK1 is involved in different types of miRNA function under the same mechanisms, using various kinds of miRNAs.

We found that RACK1 interacts with KSRP particularly when Dicer is overexpressed. KSRP is a key mediator of AU-rich element-containing mRNA degradation [27], [28] that has also been reported to bind to and promote the maturation of a subset of miRNA precursors [29]. In our study, however, maturation of miRNAs examined was preserved and the transfected synthetic miRNAs functioned appropriately in RACK1-knockdown cells. Moreover, levels of miRNAs in Ago2-containing complexes were decreased in RACK1-knockdown cells. Based on these observations, we speculate that RACK1 is only involved in the recruitment of mature miRNAs to RISCs from miRNA maturation to the point at which they exert their effects in vivo. Although the precise mechanisms by which RACK1 contributes to the recruitment of miRNAs to RISCs and the biological significance of its binding to KSRP remains to be determined, these results suggest that RACK1 may regulate miRNA function as a component of a KSRP complex and during the maturation of miRNAs and their recruitment to RISCs.

Translationally repressed mRNA accumulates in discrete cytoplasmic foci known as p-bodies [24] or in another class of cytoplasmic aggregates, stress granules [32]. Although RACK1 has been reported to mediate stress granule formation [33], no change in the localization of p-bodies or expression of the p-body marker GW182 was detected in RACK1-knockdown cells in the present study. Additionally, no interaction between p-bodies and RACK1 was observed. We thus conclude that RACK1 does not produce p-body-related effects in the miRNA pathway.

Retroviral insertion-mediated random gene disruption screening in this study identified several genes involved in miRNA function. Functional genomics approaches involving RNAi libraries have recently been used to identify genes involved in certain functional pathways [34]–[37]. However, RNAi libraries may not be suitable for identifying genes involved in miRNA- or siRNA-related pathways, because they affect the pathways being examined. Thus, random gene disruption, as used here, may be a useful alternative option for the functional screening of such genes.

While identified genes here did not include well-known components of the miRNA pathway such as Dicer and Ago2, these genes may be critical for cell survival, which would make their selection by survival screening impossible. Alternatively, it may be that the screening required a greater number of cells. Nonetheless, we newly identified six genes that may potentially be involved in miRNA function. Of the genes identified, two, RPS23 and RPL11, are ribosome-related molecules, encoding 40S and 60S subunit proteins, respectively. In view of the fact that one of RACK1's roles is as a mediator of the synthesis of 80S ribosomes (composed of 40S and 60S subunits) [18], these results may suggest the importance of the ribosomal machinery in miRNA function.

Consistent with recent evidence linking global microRNA depletion with oncogenesis [12], [30], RACK1 expression was found to be reduced in HCC. Because RACK1 staining in non-cancerous liver tissues was stronger than that in other tissues, the involvement of RACK1 in physiological functions may be more relevant in the liver. These results suggest that the miRNA functional impairment plays an important role in oncogenesis in the liver and/or the maintenance of oncogenicity in HCC, similar to the deficiency of miRNA expression [11].

## Materials and Methods

### Cell culture

Huh7 and PLC/PRF/5 cells were obtained from the Japanese Collection of Research Bioresources (JCRB, Osaka, Japan). 293T cells were obtained from the American Type Culture Collection (ATCC, Rockville, MD). Cells were maintained in Dulbecco's modified Eagle's medium (DMEM) supplemented with 10% fetal bovine serum (FBS). Stable cell lines derived from Huh7 and PLC/PRF/5 cells were established by retroviral or lentiviral infection. Clones were selected through the addition of 6 µg/mL puromycin, 400 µg/mL hygromycin, or 5 µg/mL blasticidin to the culture medium, unless otherwise specified.

### Random gene disruption

The use of a pDisrupt retroviral vector carrying a blasticidin resistance gene for random gene disruption has been described previously [13]. Retroviruses were produced by transfection of the retroviral vector into virus-packaging Platinum A cells (Orbiden, San Diego, CA). Then, 48 h after transfection, the supernatants were harvested and viruses collected. To prepare reporter cell lines, Huh7 cells were first transfected with pBSII-Hygro and selected on hygromycin. To avoid random integration in polyclonal cells, single clones were picked and expanded. The selected clones were then infected with MIR122-puro lentiviruses, which express a miR122 precursor and a puromycin resistance gene, and selected on puromycin. Again, several individual clones were isolated and expanded separately to avoid random integration. Using selected Huh7-pBSII-Hygro-miR122 cells and parental Huh7-pBSII-Hygro cells, titration of the hygromycin concentration required for total cell killing was performed to determine the clone showing the widest differences in hygromycin concentration (i.e., the clone in which expression of the hygromycin resistance gene was most effectively suppressed by miR122). Next, this clone was infected with retroviruses at low multiplicities of infection (∼0.01) to achieve random gene disruption and then treated with blasticidin. Blasticidin-resistant cells carry viruses inserted in functional genes (otherwise, the blasticidin resistant gene cannot be expressed). Then, hygromycin was added to select hygromycin resistant cells, which potentially have impaired miR122 function as a result of gene disruption (which itself results in impaired suppression of hygromycin resistance gene expression). Such clones were picked and 3′-rapid amplification of cDNA ends (RACE) analysis of the mRNAs fused with the blasticidin gene used to identify the disrupted gene in each clone.

### 3′-RACE

We infected ∼106 Huh7-pBS-Hygro-miR122 cells and obtained ∼104 blasticidin-resistant clones. After selection, ten surviving clones were obtained. The endogenous gene sequence fused with the blasticidin gene was amplified by 3′-RACE. Total RNA was isolated and reverse transcribed using an RT primer (5-CCA GTG AGC AGA GTG ACG AGG ACT CGA GCT CAA GC (T)_17_ -3′). A nested PCR was performed using primers P0/Q0 (5′-GGT GTC GAC AGG TGC TTC TC-3′/5′-CCA GTG AGC AGA GTG ACG-3′) and P1/Q1 (5′-CTG GGA TCA AAG CGA TAG TG-3′/5′-GAG GAC TCG AGC TCA AGC-3′). P0 and P1 anneal to sequences in the blasticidin resistance gene and Q0 and Q1 to the anchor sequences of the RT primer. The PCR fragments were finally subcloned into a TA-cloning vector (Invitrogen, Carlsbad, CA) and sequenced.

### Plasmids

A pBSII-Hygro plasmid containing miR122REs in its 3′-UTR was generated from pGL4-Hygro, purchased from Promega (Madison, WI). Synthetic oligonucleotides containing two tandem miR122-responsive elements were annealed and inserted in the 3′-UTR of this plasmid's hygromycin resistance gene (at the PmeI site). The primer used was 5′-AAA CAC *AAA CAC CAT TGT CAC ACT CCA* AAT TAC *AAA CAC CAT TGT CAC ACT CCA* CTC GAG-3′ (miR122-responsive sequences are shown in italics). Next, the gene cassette (containing an SV40 promoter, the hygromycin resistance gene, the miR122 responsive elements and polyA sequences) was excised using BamHI and SalI, and was inserted into pBlueScript II at the same restriction sites. pMIR-122-puro was constructed by replacing the eGFP gene of pMIRH122PA (System Biosciences, Mountain View, CA) at the FseI site by a puromycin resistance gene, which was PCR-amplified using pCDH-EF1α-puro (System Biosciences) as a template. The following primers were used: 5′-GGC CGG CCG CAT GAC GAG TAC AAG CCC AC-3′ and 5′-GGC CGG CCT CAG GCA CCG GGC TTG CGG GT-3′. pcDNA3-myc-RACK1 was used to achieve RACK1 overexpression. Two constructs targeting different RACK1 sequences, pSIH-H1-RACK1shRNA and pSuper.retro-RACK1 shRNA (both provided by Prof. Takekawa [33]), were used to achieve RACK1 knockdown. pSIH-H1-GFP shRNA was constructed as a control, as described previously [38]. To determine the effect of miR-122 on natural gene targets, a reporter plasmid (CatA-Luc) with natural 3′-UTR sequences of the CATT1 (cationic amino-acid transporter (CAT-1)) gene containing three predicted miR-122 binding sites was used. This plasmid was provided by Prof. W. Filipowicz [22]. Reporter plasmids used to analyze miRNA function were constructed by inserting annealed synthetic primers containing two tandem sequences complementary to each miRNA into the 3′-UTR of the firefly luciferase gene at the FseI site, driven by the CMV promoter (pGL3-basic; Promega). The following primers were used: miR-122, 5′-ACA AAC ACC ATT GTC ACA CTC CAA CTT CAC CCA ACC ATT GTC ACA CTC CAC TCG AGC CGG-3′; miR-140-5p, 5′-CTA CCA TAG GGT AAA ACC ACT GAA TTC TAC CAT AGG GTA AAA CCA CTG CTC GAG CCG G-3′; miR-185, 5′-TCA GGA ACT GCC TTT CTC TCC AAA TTT CAG GAA CTG CCT TTC TCT CCA CTC GAG CCG G-3′. The flag-tagged Dicer-expressing plasmid has been described previously [39]. MiR precursor overexpressing plasmids were purchased from System Biosciences.

### Transfection

Plasmid transfection was performed using FuGENE6 (Boehringer Mannheim, Mannheim, Germany) according to the manufacturer's protocol [40]. Synthetic mature miRNA oligonucleotides (miNatural, CosmoBio, Tokyo, Japan) were transfected using TransMessenger Transfection Reagent (Qiagen, Hilden, Germany).

### Antibodies

The following antibodies were used in Western blotting analyses: anti-Dicer (SAB4200087; Sigma, St. Louis, MO), anti-TRBP2 (SAB4200087; Sigma), anti-β-actin (A5316; Sigma), anti-Ago2 (#015-22031; Wako, Osaka, Japan), anti-Gemin3 (DDX20) (SC-57007; Santa Cruz Biotechnology, Santa Cruz, CA), anti-Gemin4 (H00050628; Abnova, Taipei, Taiwan), anti-eIF4E (#9742; Cell Signaling Technology, Danvers, MA), anti-eIF6 (D16E9; Cell Signaling Technology), anti-KSRP (A302-021A; Bethyl, Montgomery, TX), anti-GW182 (MBL Nagoya, Japan), anti-Drosha (#3364; Cell Signaling Technology), anti-DGCR8 (SAB 4200088; Sigma), anti-Ago1 (clone1F2; Wako, Osaka, Japan), anti-Ago3 (SAB2104518; Sigma), anti-Ago4 (SAB2104338; Sigma), anti-Lamin B (SC-20682; Santa Cruz Biotechnology), anti-GAPDH (clone 3C2; Abnova), and anti-myc (Santa Cruz Biotechnology). An Atlas anti-RACK1 antibody was used in immunohistochemical analyses (HPA021676; Sigma).

### Western blotting

Cell extract protein concentrations were measured using a DC Protein Assay Kit (Bio-Rad, Hercules, CA). Total protein (30 µg) was resolved by sodium dodecyl sulfate- polyacrylamide gel electrophoresis (SDS-PAGE) and then transferred to Hybond-P polyvinylidene difluoride (PVDF) membranes (Amersham Pharmacia Biotech, Little Chalfont, Buckinghamshire, UK). Membranes were sequentially incubated with primary antibody and horseradish peroxidase-conjugated secondary antibody. Bound antibody was detected using ImmunoStar reagents (Wako) [41].

### Cell fractionation

To separate cytoplasmic and nuclear protein fractions, a ProteoExtract subcellular fractionation kit (Calbiochem-EMD Biosciences, San Diego, CA) was used according to the manufacturer's protocol. To confirm the identities of the subcellular fractions, Lamin B was blotted for the nuclear fraction and GAPDH for the cytoplasmic fraction.

### Immunocytochemistry

To determine the localization of p-bodies, cells growing on two-well chamber slides were fixed with 4% paraformaldehyde and permeabilized with 0.5% Triton-X100. Fixed cells were then probed with an anti-GW182 antibody for 1 h after blocking with 5% normal goat serum for 30 min. They were then treated with an Alexa Fluor 555-conjugated goat anti-rabbit secondary antibody (Invitrogen) for 30 min. Slides were mounted using VectaShield with DAPI (Vector Labs, Burlingame, CA). Numbers of p-bodies in the cells were determined in three independent views, and the average number or p-bodies per cell calculated. Similar procedures were applied to determine KSRP and Ago2 intracellular localization, except that anti-KSRP and anti-Ago2 were used as first antibodies, and Alexa Fluor 555-conjugated goat anti-rabbit antibody and Alexa Fuor 488-conjugated anti-mouse antibody were used as secondary antibodies.

### Immunohistochemistry

Tissue arrays containing multiple organ carcinoma tissues and matched adjacent non-cancerous tissues (1.5 cm apart; #MC501 and #MC962) were purchased from US Biomax (Rockville, MD). Slides were incubated at 65°C for 1 h and deparaffinized. Endogenous peroxidase activity was blocked through incubation in 3% hydrogen peroxide buffer for 30 min. Antigen retrieval was achieved by incubating the slides at 89°C in 10 mM sodium citrate buffer (pH 6.0) for 30 min. To minimize non-specific background staining, slides were blocked in 5% normal goat serum (Dako, Glostrup, Denmark) for 10 min at room temperature. A primary anti-RACK1 antibody or anti-KSRP antibody, diluted 1∶50 in Antibody Diluent (Dako), was applied for 1 h at room temperature. Slides were then incubated with an anti-mouse horseradish peroxidase-conjugated secondary antibody (Nichirei Bioscience, Tokyo, Japan) for 1 h. Bound antibody was visualized by incubation in 3,3′-diaminobenzidine (Nichirei Bioscience; diluted in buffered substrate) for 5 min. The slides were finally counterstained with hematoxylin, dehydrated with ethanol, and mounted using Clarion mounting medium (Biomeda, Foster City, CA).

### Luciferase assay

Luciferase activity were measured using a Dual Luciferase Reporter Assay System (Promega) in conjunction with a Lumat LB9507 luminometer (EG&G Berthold, Bad Wildbad, Germany). pRL-TK, a control plasmid carrying the Renilla reniformis (sea pansy) luciferase gene, driven by the herpes simplex virus thymidine kinase promoter (Toyo Ink, Tokyo, Japan), was used as an internal control. Relative luciferase signals were calculated by dividing firefly luciferase activity by that of the internal control (sea pansy luciferase) (unless otherwise specified). All experiments were performed at least twice (each time in triplicate).

### Lentiviruses and transduction

293T cells were transfected with pPACKH1 Packaging Plasmid Mix (System Biosciences) and pCDH (as a negative control), pSIH-H1-RACK1shRNA, or pMIR122-puro. After 2 days, supernatants were collected and the viruses concentrated using PEG-it Virus Precipitation Solution (System Biosciences) according to the manufacturer's protocol. Lentiviral particles expressing shRNAs specific for Ago2 (sc-44409) and KSRP (sc-44831) were purchased from Santa Cruz Biotechnology. Polybrene (Millipore, Billerica, MA) was used in lentiviral particle infection.

### miRNA isolation, quantitation, and RIP assay

A Mir-X miRNA qRT-PCR SYBR Kit (Clontech, Mountain View, CA) was used in accordance with the manufacturer's protocol to measure levels of different miRNAs in cells. Total RNA was isolated from cells and tissues using the TRIzol reagent (Invitrogen). Levels of U6 snRNA were used in the normalization of cellular miRNA levels. Relative expression levels were calculated by the ΔΔCT method: ΔΔCT  =  ΔCTmiRNA – ΔCTU6. To purify miRNAs from Ago2-containing RISC-associated miRNP complexes, miRNA fractions were isolated using the Human Ago2 MicroRNA Isolation Kit (Wako), using antibodies raised against Ago2. The following primers were used in quantitative PCR analyses: miR122, 5′-TGG AGT GTG ACA ATG GTG TTT G-3′; and miR-185, 5′-TGG AGA GAA AGG CAG TTC CTG A-3′.

### Immunoprecipitation

Huh7 cells were transfected with pcDNA3 (negative control) or myc-tagged RACK1-expressing plasmid with or without Dicer-expressing plasmid. When required, cell lysates were incubated at room temperature with RNase A (10 µg/mL; Promega) for 30 min. Cell extracts were prepared using immunoprecipitation buffer (50 mM Tris-HCl (pH 7.5), 150 mM NaCl, 0.1% NP-40, 1 mM EDTA, 0.25% gelatin, 0.02% sodium azide, 100 µg/mL phenylmethylsulfonyl fluoride, 1 µg/mL aprotinin). Myc-tagged RACK1 was precipitated through incubation with anti-myc agarose (#SC-40; Santa Cruz Biotechnology) for 8 h. Anti-KSRP antibodies and Protein A/G Sepharose (#SC-2003; Santa Cruz Biotechnology) were used for endogenous KSRP precipitation. To immunoprecipitate endogenous RACK1 protein, anti-RACK1 antibodies and Protein A/G Sepharose were used. After being washed four times, the precipitated proteins were analyzed by Western blotting using the indicated antibodies. Five percent of each cell lysate was used as “input.”

### Statistical analysis

Statistically significant differences between groups were identified using Student's t-test (when variances were equal) or Welch's t-test (when variances were not equal).

## Supporting Information

Figure S1 **miRNA maturation was not impaired in RACK1-knockdown cells.** Total RNA was isolated from control and RACK1-knockdown cells. Levels of mature miR22, miR140-5p, miR140-3p, and miR185, all of which are relatively abundant miRNAs in liver cells, were measured and normalized to the level of U6 snRNA. Relative ratios were calculated by adjusting the value for each miRNA in control cells to 1. Data represent the mean ± SD of six independent experiments.
(TIF)

Figure S2 **Synthetic mature miRNAs were functional in Ago2-kcnockdown cells.** **A,** Ago2-knockdown (Ago2 KD) Huh7 cells were established by shRNA-expressing lentiviral infection. **B,** Artificial synthetic miRNA oligonucleotides function normally in Ago2-knockdown Huh7 cells. Control and Ago2-knockdown (Ago2 KD) cells were transfected with miR122 or miR185 reporter plasmids with corresponding synthetic mature miRNA oligonucleotides. Values were normalized to those obtained from cells transfected with control synthetic oligonucleotides, which were set to 1. Data represent the mean ± SD of three independent experiments. *, p<0.05.
(TIF)

Figure S3 **RACK1 binds to KSRP.** Huh7 cells were transiently transfected with a control vector or a myc-tagged RACK1-expressing plasmid, with or without a Flag-tagged Dicer-expressing plasmid. Endogenous KSRP was immunoprecipitated using anti-KSRP with Protein A/G Sepharose. Normal mouse IgG was used as a control for immunoprecipitation. Co-precipitated proteins were blotted using antibodies against myc tag. Five percent of the total cell lysates was loaded as “input.” Representative results from two independent experiments are shown.
(TIF)

Figure S4 **Coimmunoprecipitation of endogenous KSRP and Ago2 with myc-RACK1 is insensitive to RNase A treatment.**Huh7 cells were transiently transfected with a control vector or a myc-tagged RACK1-expressing plasmid. Myc-tagged RACK1 was immunoprecipitated using anti-myc antibody conjugated to agarose. Normal mouse IgG was used as a control for immunoprecipitation. Coprecipitated proteins were blotted using antibodies against the indicated proteins. Five percent of the total cell lysate was loaded as “input.” Representative results from two independent experiments are shown.
(TIF)

Figure S5 **RACK1 may be involved in the recruitment of mature miRNAs to the RISC.** Mature miRNA levels were measured in RNA samples isolated from Ago2-containing complexes from control Huh7 (NC) cells and RACK1-knockdown (siRACK1) cells. miRNA levels were calculated as relative ratios. Data represent the mean ± SD of six independent experiments. * p<0.05.
(TIF)

Figure S6 **KSRP is not required for RISC activity. A,** KSRP-knockdown Huh7 cells were established by shRNA-expressing lentiviral infection. **B,** Artificial synthetic miRNA oligonucleotides function normally in KSRP-knockdown Huh7 cells. Control and Ago2-knockdown cells were transfected with miR122 or miR185 reporter plasmids with corresponding synthetic mature miRNA oligonucleotides. Values were normalized to those obtained from cells transfected with control synthetic oligonucleotides, which were set to 1. Data represent the mean ± SD of three independent experiments. *, p<0.05.
(TIF)

Figure S7 **KSRP does not bind with Ago2.** Endogenous KSRP was immunoprecipitated using anti-KSRP with Protein A/G Sepharose. Normal rabbit IgG was used as a control for immunoprecipitation. Co-precipitated proteins were blotted using antibodies against Ago2.
(TIF)

Figure S8 **The levels of KSRP expression are high in liver tissues. A,** Immunohistochemical analysis of KSRP protein expression in hepatocellular carcinoma (HCC) and non-cancerous surrounding tissues. Lower panels: magnified images of the highlighted regions in the corresponding upper panels. Three representative cases are shown. For a comparison, the sections used here were almost the same parts used for determining RACK1 expression in Fig. 5. Scale bar, 500 µm. **B,** Relatively low expression levels of KSRP both in colon carcinoma tissues (right images) and non-cancerous surrounding tissues (left). Two representative cases are shown. Again, for a comparison, the sections used here were almost the same parts used for determining RACK1 expression in Fig. 5. Scale bar, 500 µm.
(TIF)
