# Supplementary figures and images for: Receptor for Activated Protein Kinase C: Requirement for Efficient MicroRNA Function and Reduced Expression in Hepatocellular Carcinoma
Source: PLoS One. 2011 Sep 15;6(9):e24359. doi: 10.1371/journal.pone.0024359 (PMC3174171; doi:10.1371/journal.pone.0024359)

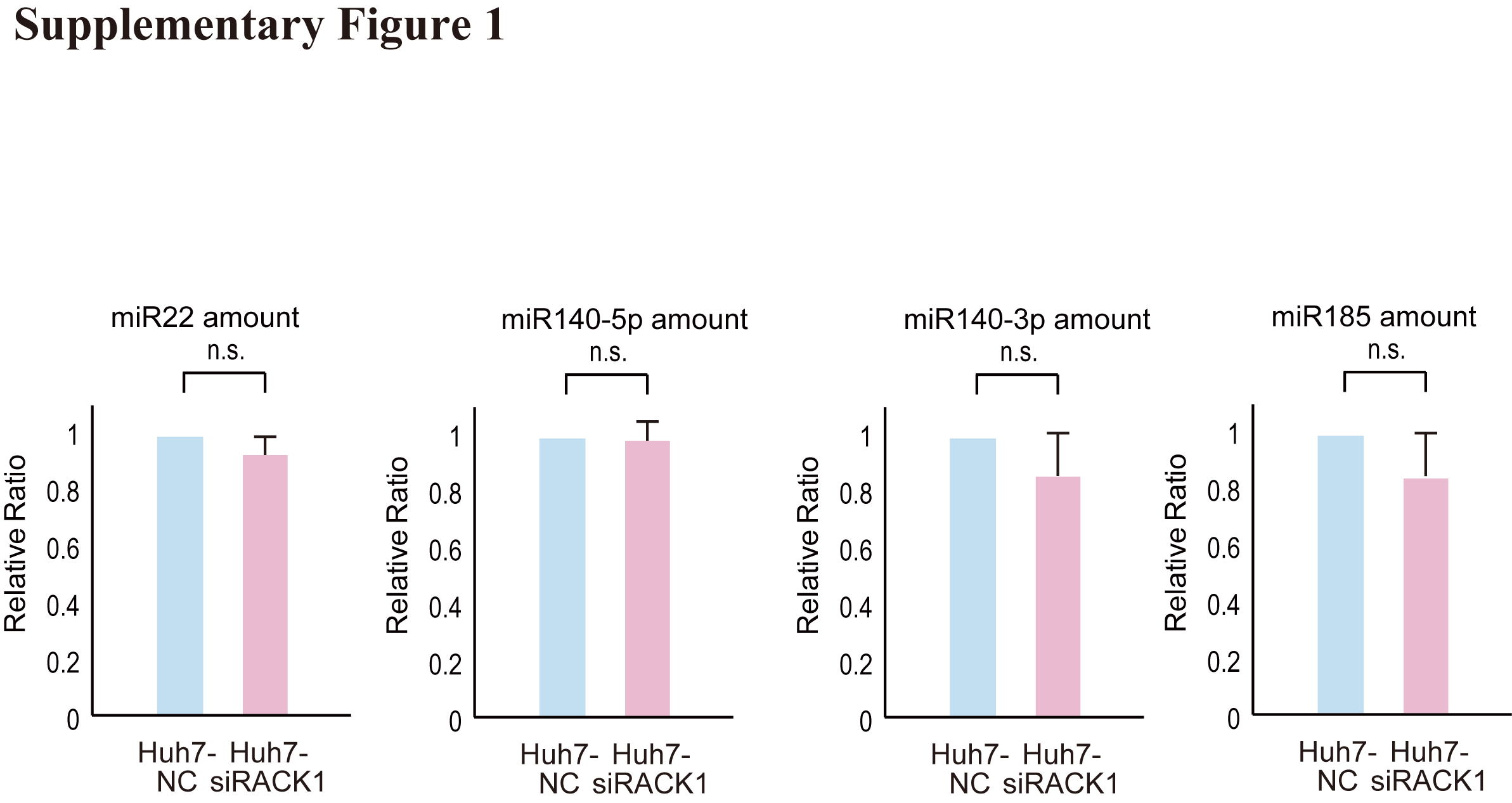

Supplement: Figure S1 — miRNA maturation was not impaired in RACK1-knockdown cells. Total RNA was isolated from control and RACK1-knockdown cells. Levels of mature miR22, miR140-5p, miR140-3p, and miR185, all of which are relatively abundant miRNAs in liver cells, were measured and normalized to the level of U6 snRNA. Relative ratios were calculated by adjusting the value for each miRNA in control cells to 1. Data represent the mean ± SD of six independent experiments. (TIF) [file pone.0024359.s001.tif]

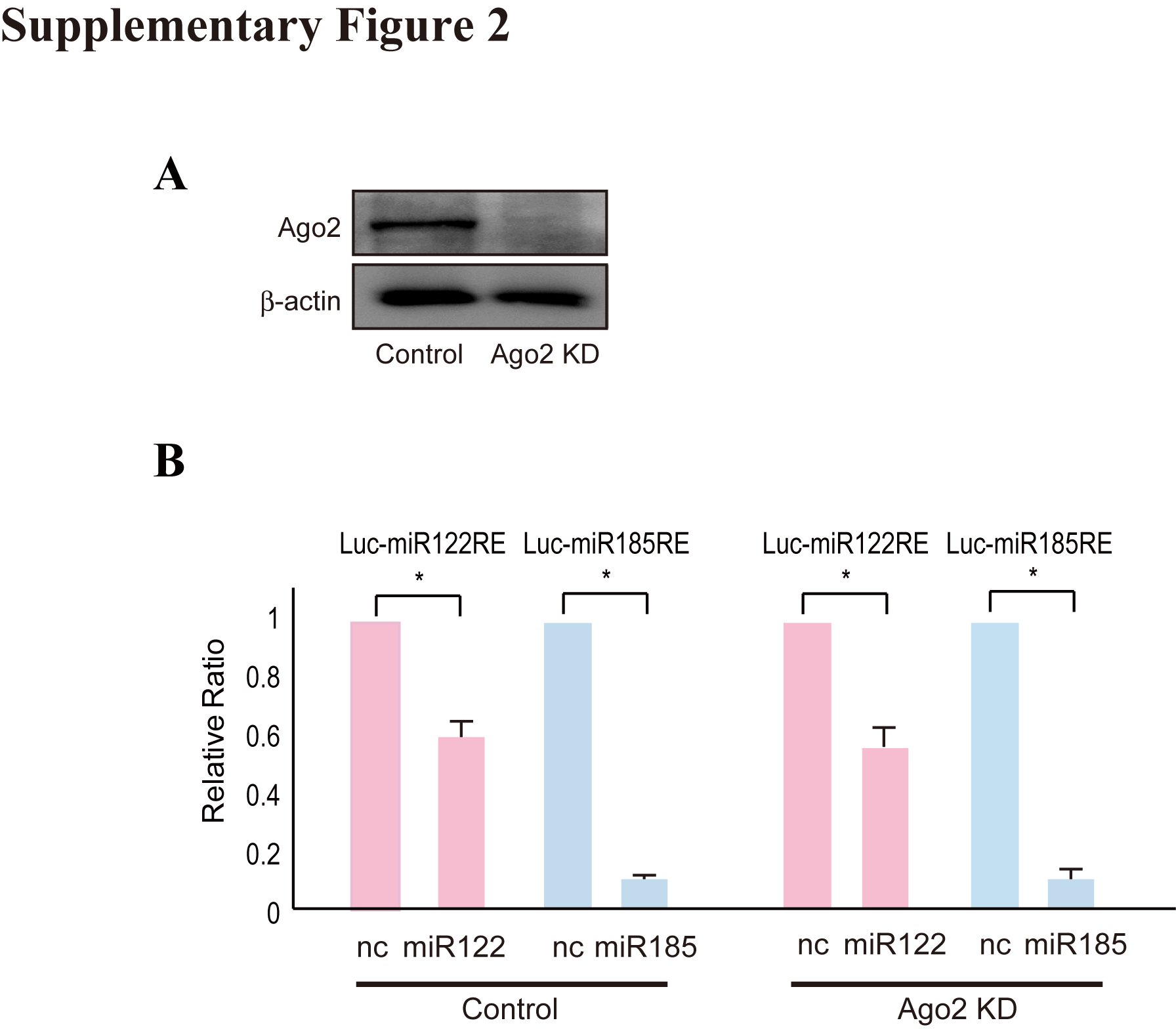

Supplement: Figure S2 — Synthetic mature miRNAs were functional in Ago2-kcnockdown cells. A, Ago2-knockdown (Ago2 KD) Huh7 cells were established by shRNA-expressing lentiviral infection. B, Artificial synthetic miRNA oligonucleotides function normally in Ago2-knockdown Huh7 cells. Control and Ago2-knockdown (Ago2 KD) cells were transfected with miR122 or miR185 reporter plasmids with corresponding synthetic mature miRNA oligonucleotides. Values were normalized to those obtained from cells transfected with control synthetic oligonucleotides, which were set to 1. Data represent the mean ± SD of three independent experiments. *, p<0.05. (TIF) [file pone.0024359.s002.tif]

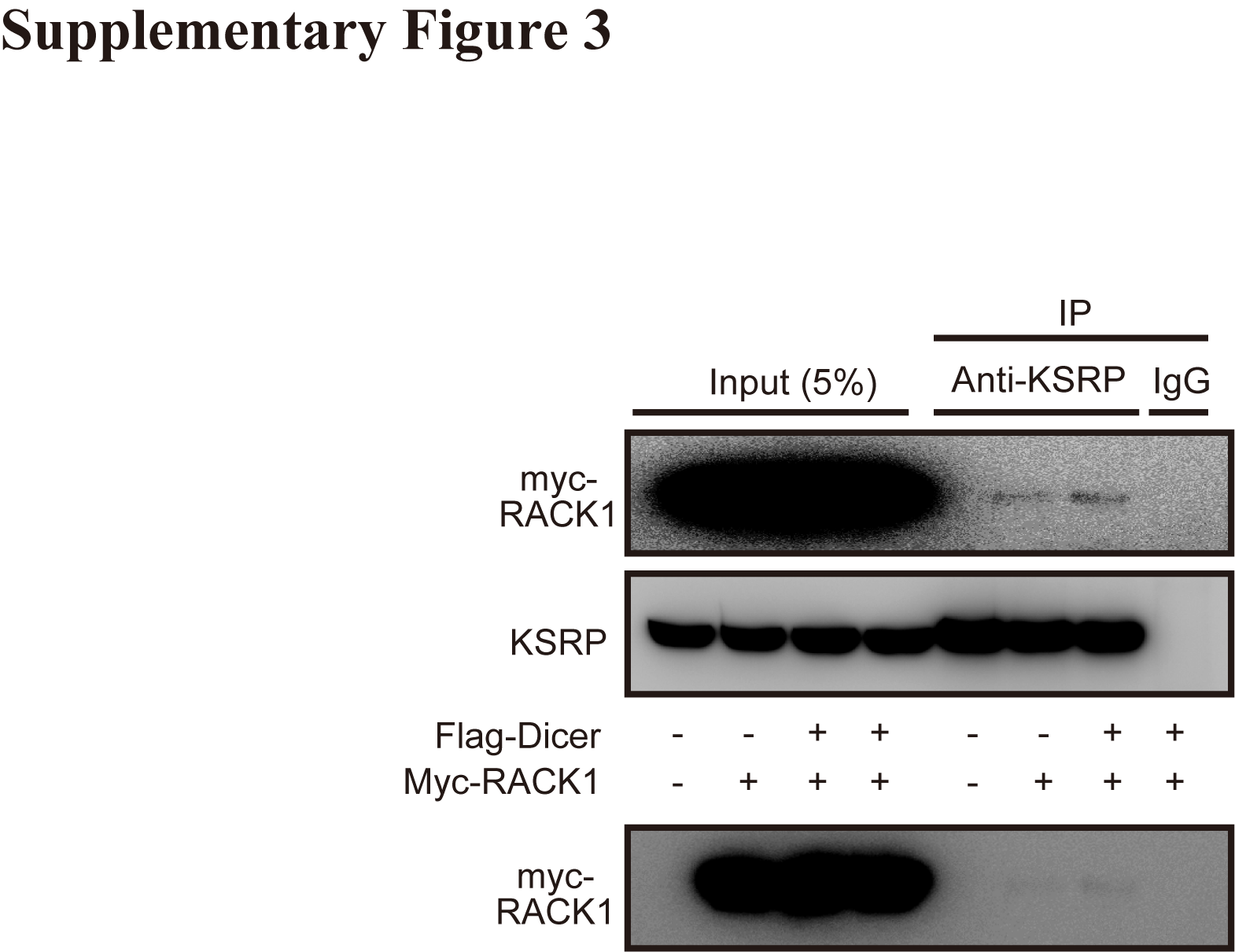

Supplement: Figure S3 — RACK1 binds to KSRP. Huh7 cells were transiently transfected with a control vector or a myc-tagged RACK1-expressing plasmid, with or without a Flag-tagged Dicer-expressing plasmid. Endogenous KSRP was immunoprecipitated using anti-KSRP with Protein A/G Sepharose. Normal mouse IgG was used as a control for immunoprecipitation. Co-precipitated proteins were blotted using antibodies against myc tag. Five percent of the total cell lysates was loaded as “input.” Representative results from two independent experiments are shown. (TIF) [file pone.0024359.s003.tif]

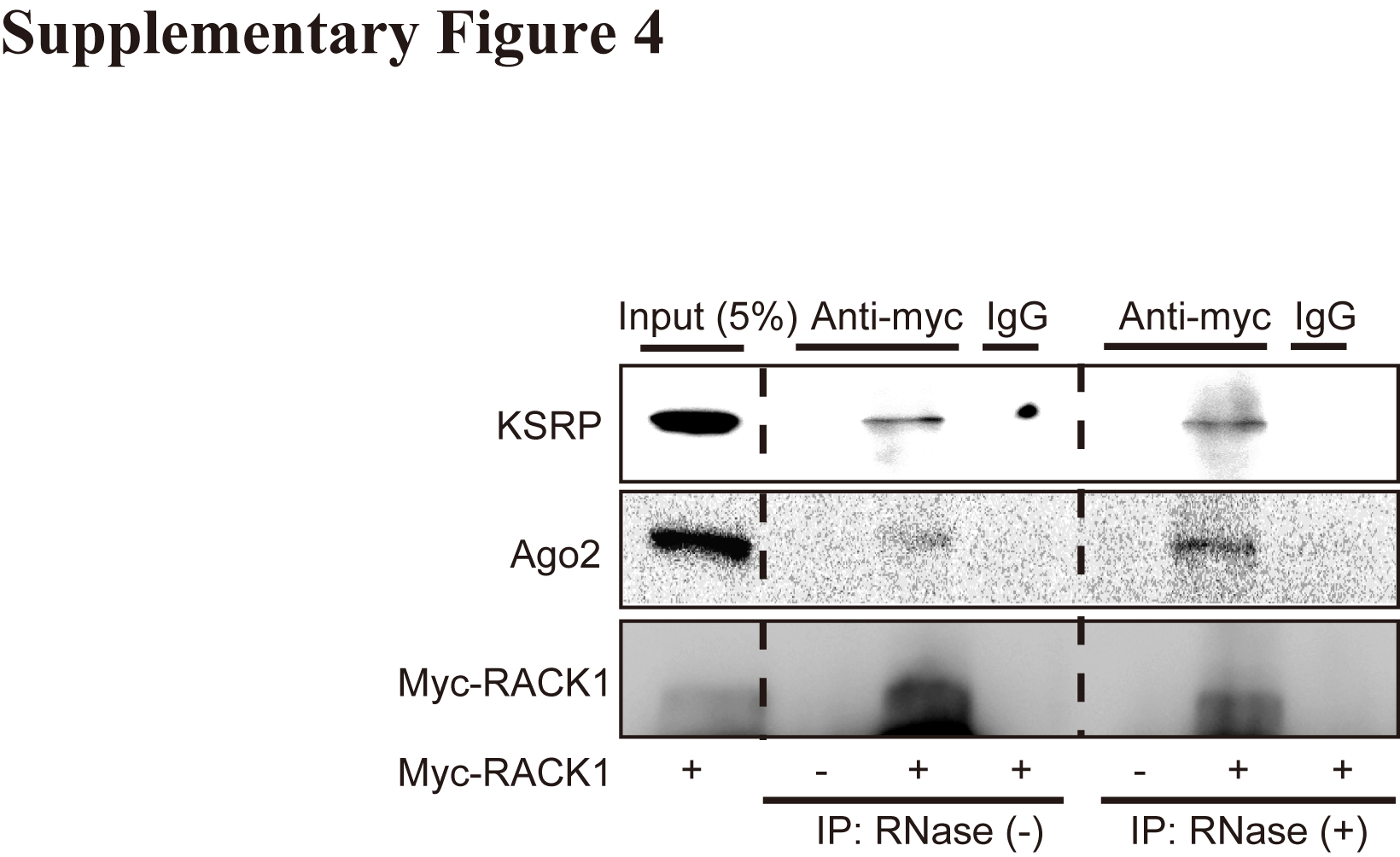

Supplement: Figure S4 — Coimmunoprecipitation of endogenous KSRP and Ago2 with myc-RACK1 is insensitive to RNase A treatment.Huh7 cells were transiently transfected with a control vector or a myc-tagged RACK1-expressing plasmid. Myc-tagged RACK1 was immunoprecipitated using anti-myc antibody conjugated to agarose. Normal mouse IgG was used as a control for immunoprecipitation. Coprecipitated proteins were blotted using antibodies against the indicated proteins. Five percent of the total cell lysate was loaded as “input.” Representative results from two independent experiments are shown. (TIF) [file pone.0024359.s004.tif]

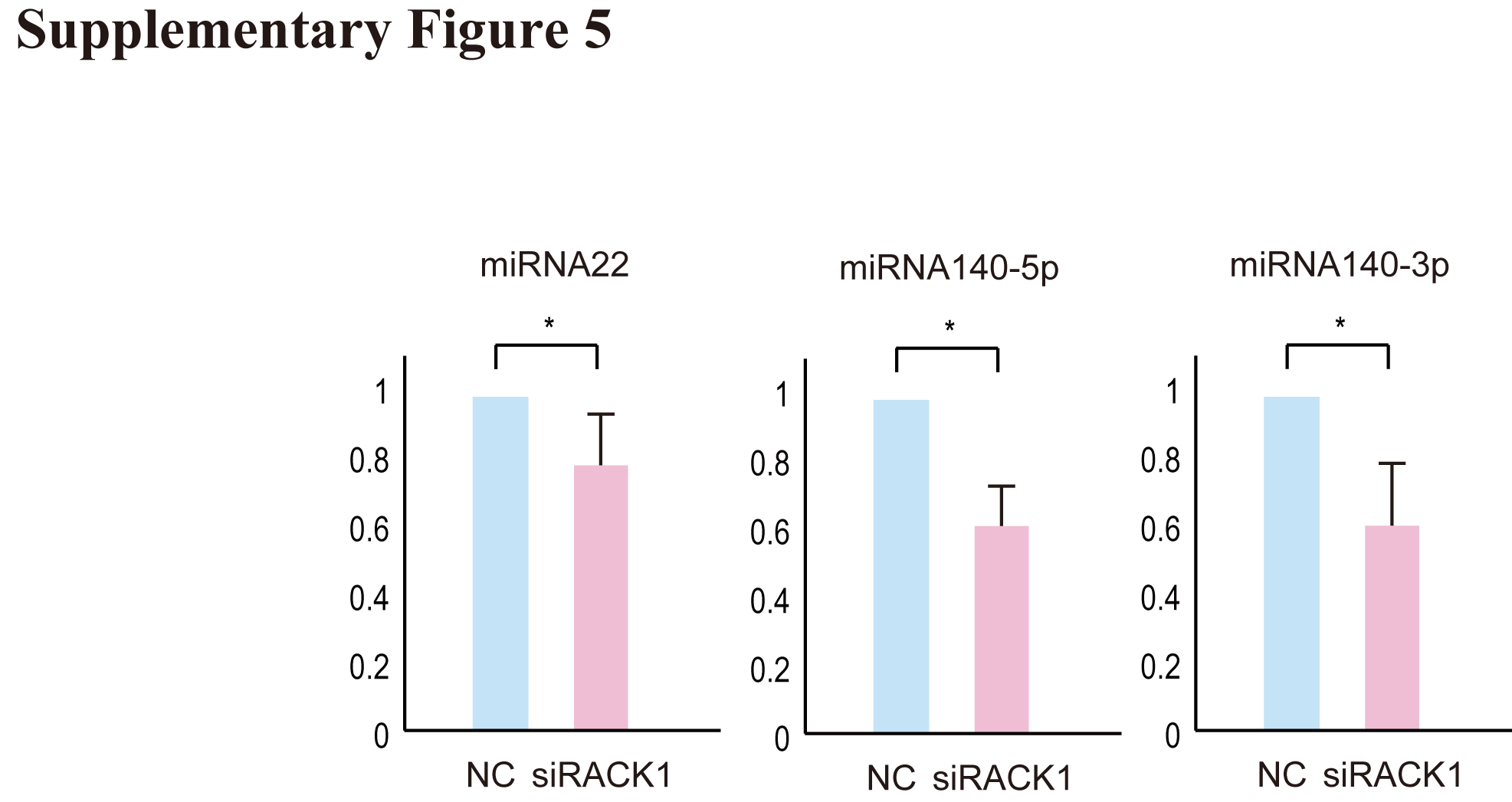

Supplement: Figure S5 — RACK1 may be involved in the recruitment of mature miRNAs to the RISC. Mature miRNA levels were measured in RNA samples isolated from Ago2-containing complexes from control Huh7 (NC) cells and RACK1-knockdown (siRACK1) cells. miRNA levels were calculated as relative ratios. Data represent the mean ± SD of six independent experiments. * p<0.05. (TIF) [file pone.0024359.s005.tif]

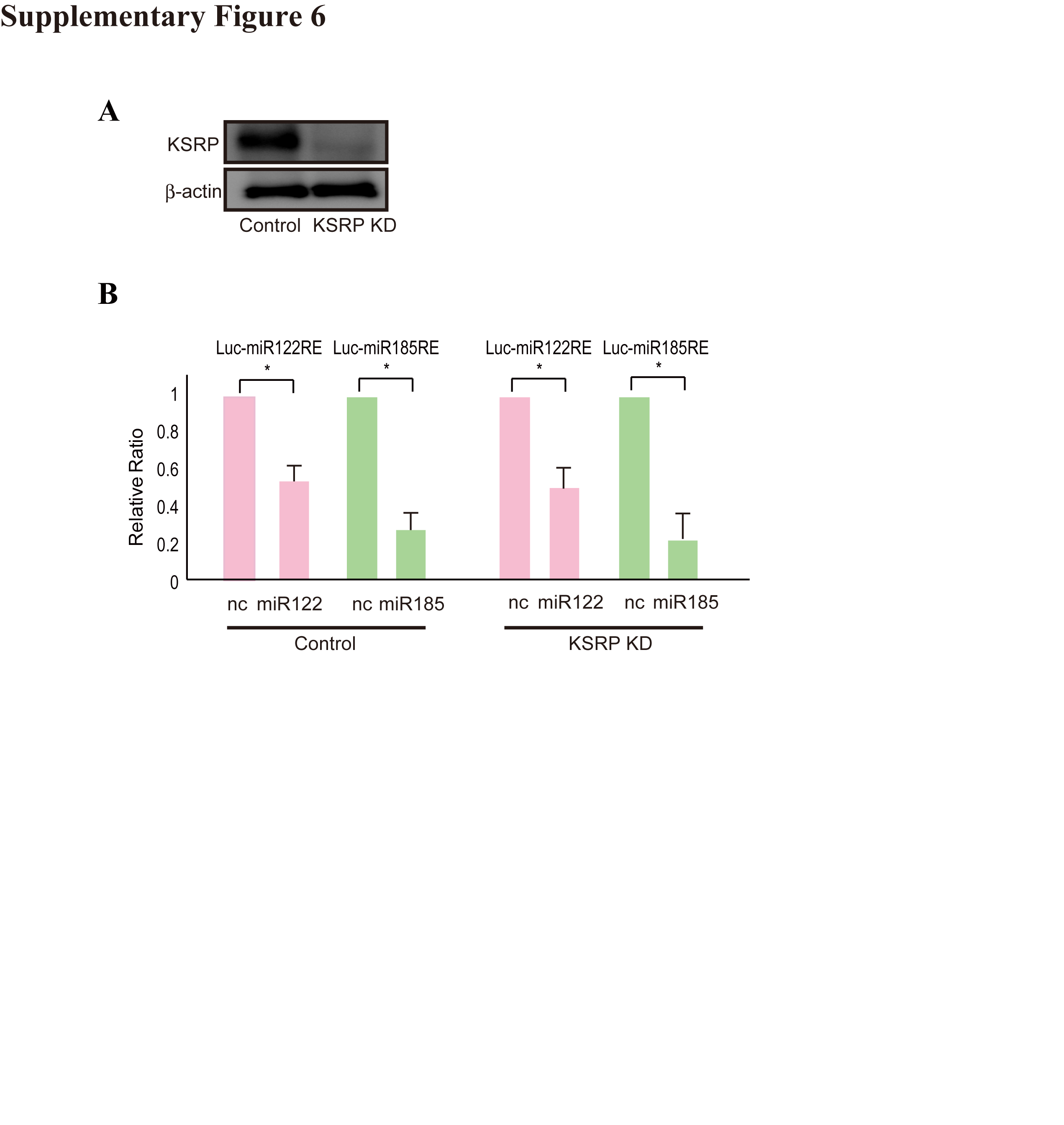

Supplement: Figure S6 — KSRP is not required for RISC activity. A, KSRP-knockdown Huh7 cells were established by shRNA-expressing lentiviral infection. B, Artificial synthetic miRNA oligonucleotides function normally in KSRP-knockdown Huh7 cells. Control and Ago2-knockdown cells were transfected with miR122 or miR185 reporter plasmids with corresponding synthetic mature miRNA oligonucleotides. Values were normalized to those obtained from cells transfected with control synthetic oligonucleotides, which were set to 1. Data represent the mean ± SD of three independent experiments. *, p<0.05. (TIF) [file pone.0024359.s006.tif]

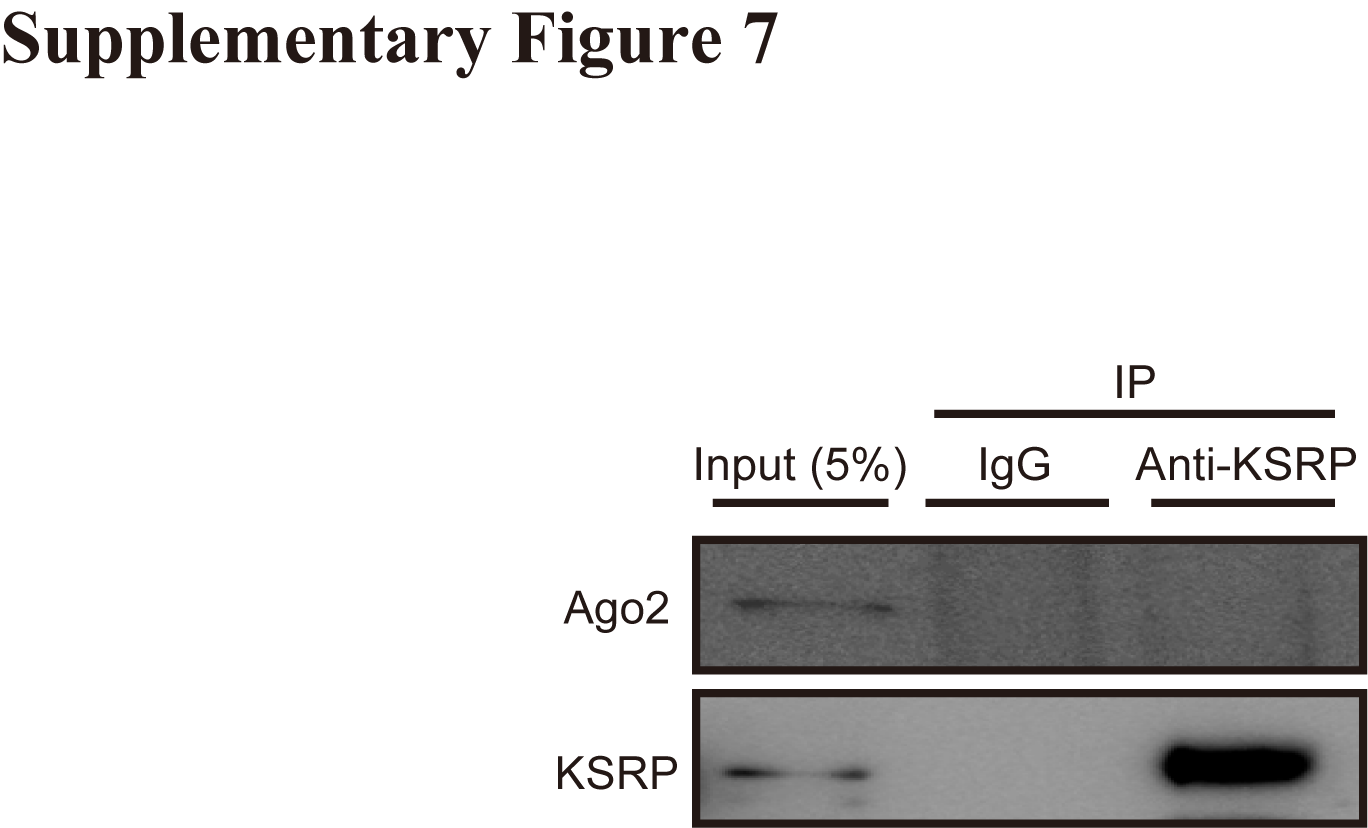

Supplement: Figure S7 — KSRP does not bind with Ago2. Endogenous KSRP was immunoprecipitated using anti-KSRP with Protein A/G Sepharose. Normal rabbit IgG was used as a control for immunoprecipitation. Co-precipitated proteins were blotted using antibodies against Ago2. (TIF) [file pone.0024359.s007.tif]

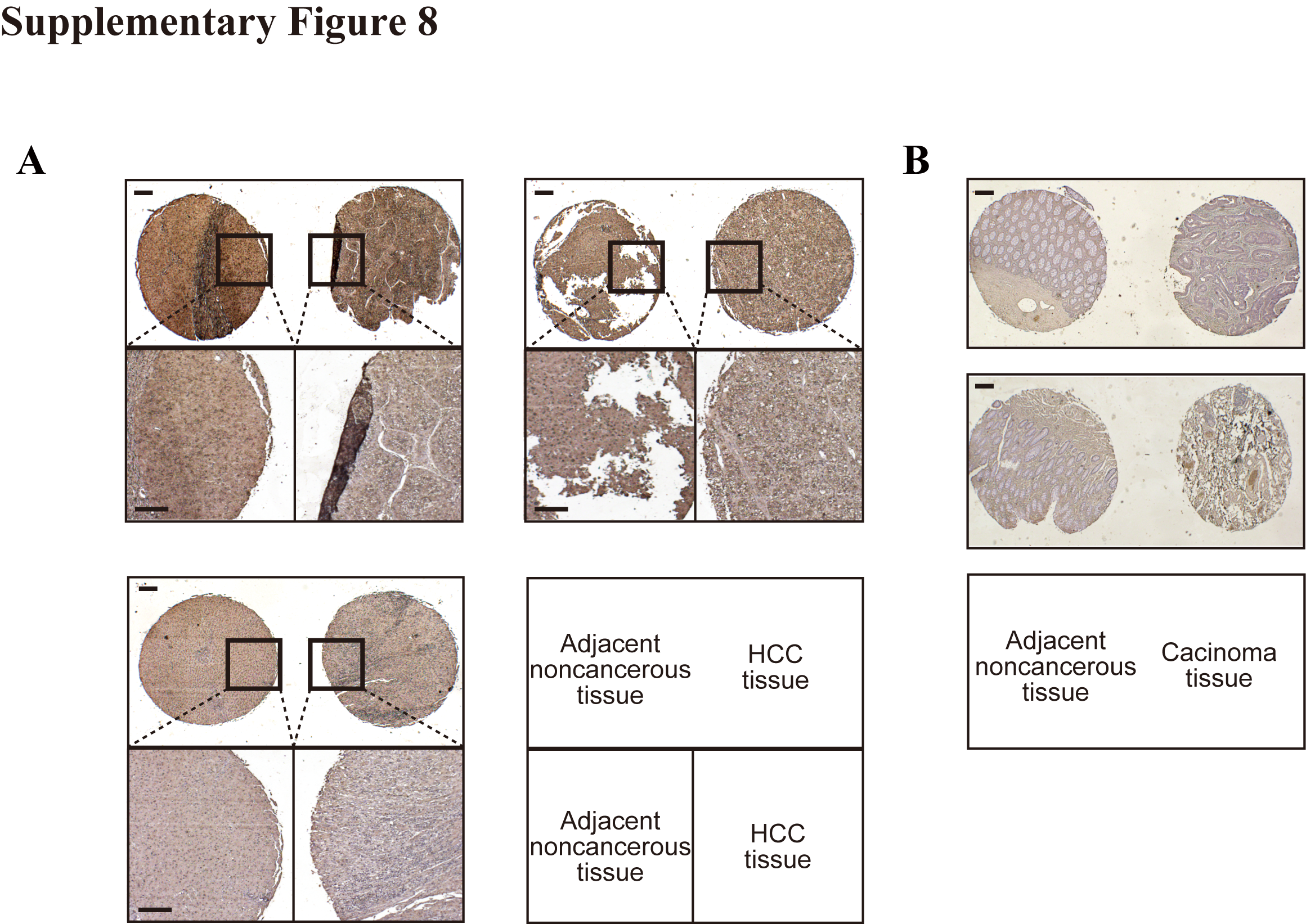

Supplement: Figure S8 — The levels of KSRP expression are high in liver tissues. A, Immunohistochemical analysis of KSRP protein expression in hepatocellular carcinoma (HCC) and non-cancerous surrounding tissues. Lower panels: magnified images of the highlighted regions in the corresponding upper panels. Three representative cases are shown. For a comparison, the sections used here were almost the same parts used for determining RACK1 expression in Fig. 5. Scale bar, 500 µm. B, Relatively low expression levels of KSRP both in colon carcinoma tissues (right images) and non-cancerous surrounding tissues (left). Two representative cases are shown. Again, for a comparison, the sections used here were almost the same parts used for determining RACK1 expression in Fig. 5. Scale bar, 500 µm. (TIF) [file pone.0024359.s008.tif]
